# Development and validation of a SEER-based prognostic nomogram for cervical cancer patients below the age of 45 years

**DOI:** 10.17305/bjbms.2020.5271

**Published:** 2021-10

**Authors:** Qunlong Liu, Wenxia Li, Ming Xie, Ming Yang, Mei Xu, Lei Yang, Bing Sheng, Yanna Peng, Li Gao

**Affiliations:** Department of Obstetrics and Gynecology, The People’s Hospital of Yingshang, Anhui, China

**Keywords:** Early-onset cervical cancer, prognostic nomogram, overall survival, cancer-specific survival, SEER

## Abstract

In this study, we established a nomogram for the prognostic prediction of patients with early-onset cervical cancer (EOCC) for both overall survival (OS) and cancer-specific survival (CSS). The Surveillance, Epidemiology, and End Results (SEER) database was used to identify 10,079 patients diagnosed with EOCC between 2004 and 2015; these cases were then randomly divided into training and validation sets. The independent prognostic factors were identified in a retrospective study of 7,055 patients from the training set. A prognostic nomogram was developed using R software according to the results of multivariable Cox regression analysis. Furthermore, the model was externally validated using the data from the remaining 3,024 patients diagnosed at different times and enrolled in the SEER database. For the training set, the C-indexes for OS and CSS prediction were determined to be 0.831 (95 % confidence interval [CI]: 0.815–0.847) and 0.855 (95 % CI: 0.839–0.871), respectively. Receiver operating characteristic (ROC) analysis has revealed that the nomograms were a superior predictor compared with TNM stage and SEER stage. The areas under the curve (AUC) of the nomogram for OS and CSS prediction in the ROC analysis were 0.855 (95 % CI: 0.847–0.864) and 0.782 (95 % CI: 0.760–0.804), respectively. In addition, calibration curves indicated a perfect agreement between the nomogram-predicted and the actual 1-, 3-, and 5-year OS and CSS rates in the validation cohort. Thus, in this study, we established and validated a prognostic nomogram that provides an accurate prediction for 3-, 5-, and 10-year OS and CSS of EOCC patients. This will be useful for clinicians in guiding counseling and clinical trial design for cervical cancer patients.

## INTRODUCTION

Cervical cancer remains the fourth most common female malignancy in the world [1,2], ranking second as the leading cause of cancer-related deaths in young women aged 20 to 39 years in 2020 [1]. The global incidence of cervical cancer is approximately 500,000 cases annually [3], and the number of new cases and deaths in China accounts for more than one-fourth that of the entire world [4]. At present, surgery, radiotherapy, chemotherapy, and immunotherapy serve as the primary treatments for this type of cancer [5]. Clinically, patients younger than 45 years old are defined as having early-onset cervical cancer. Although significant progress has been made in surgery, radiotherapy, and chemotherapy for treating cervical cancer, there remain significant differences in clinical prognosis, especially with elderly patients. Therefore, accurate prognostic markers and improved individualized treatment are needed.

At present, the tumor lymph node metastasis (TNM) staging system proposed by the American Joint Commission on Cancer (AJCC) has been widely used to predict the prognosis of various cancers. This system considers tumor invasion (T), regional lymph node (N), and distant metastasis (M) as predictors [6]. However, prognosis based on the TNM staging system remains limited and does not accurately predict prognosis. To establish an individualized treatment plan, it is necessary to consider all the risk factors related to cancer, especially for the treatment of EOCC patients.

In recent years, nomograms based on the regression coefficient of each variable integrate multiple prognostic factors and may better predict survival rate [7]. It has been used to predict the prognosis of various cancers such as gastric cancer [8] and breast cancer [9]. As a prognostic tool, nomograms can accurately predict the overall survival rate (OS) and cancer-specific survival rate (CSS) of patients, which is based on multiple clinical variables included in the calculation. In this study, we established nomograms to predict the 3-, 5-, and 10- year OS and CSS of EOCC patients, which may be deemed useful for establishing individualized treatments and improving patient outcome.

## MATERIALS AND METHODS

### Data source and patients

**FIGURE 1:**
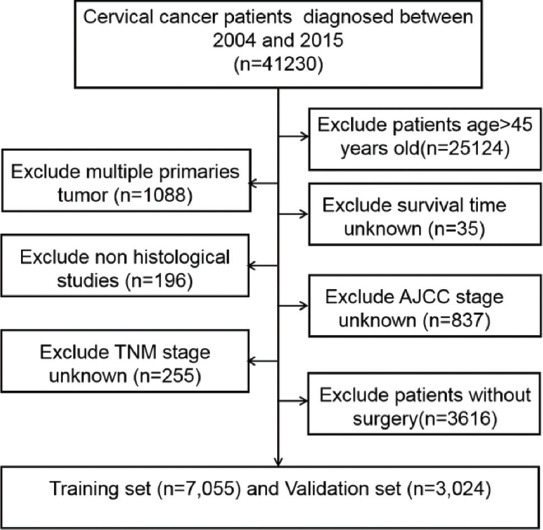
Schematic of patient screening process.

From 2004 to 2015, we adopted SEER * stat software [version 8.3.5; SEER 18 Regs Custom Data (including additional treatment fields), November 2018 sub (1975-2016 varying) database] in order to identify 10,079 eligible patients who were diagnosed with EOCC from the SEER database of the National Cancer Institute, which includes clinicopathological and individualized prognosis data. The exclusion criteria were as follows: (I) patients over 45 years old; (II) patients with multiple primary tumors; (III) unknown survival time; (IV) non-histological studies; (V) unknown AJCC stage; (VI) unknown TNM stage; and (VII) patients with no surgery. The screening scheme for the subjects is provided in Figure 1. All eligible EOCC patients were randomly assigned to a training and validation set.

### Study variables

**FIGURE S1:**
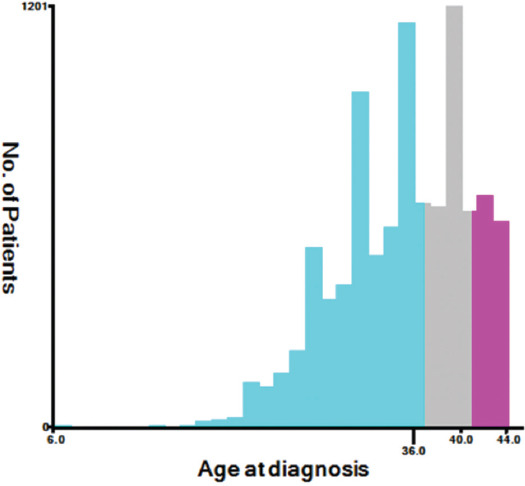
Estimation of the cut-off value for the age determined by X-tile software.

Clinical variables extracted from the SEER database included age at diagnosis, race, marital status, histological type of origin, primary tumor site, histologic type, tumor academic stage, NM stage, SEER stage, tumor size (cm), chemotherapy, and radiotherapy. The EOCC patients were then divided into three groups based on age (<37, 37–40, and >40; Fig. S1) according to the optimal cut-off value calculated by X-tile software (version 3.6.1, Yale University School of Medicine, USA). The clinical characteristics included race (white, black, and others) and marital status (married, unmarried, unknown). The tumor variables included the histological type (squamous cell carcinoma, adenocarcinoma, and others), SEER stage (localized, regional, and distant), tumor size (cm) (≤4 cm, >4 cm, and unknown), radiotherapy (no or yes), and chemotherapy (no or yes). Tumor grades I–IV were categorized as well differentiated, moderately differentiated, poorly differentiated, and undifferentiated. OS time refers to the survival time of the patient from diagnosis to any cause of death or the date at which data were deleted. The CSS time refers to the cancer-related survival time from diagnosis to death, excluding other factors. The study end point was survival (OS and CSS).

### Ethical statement

Since the clinical data in this study were collected from a publicly available database, there were no local or state ethical issues. In addition, because this retrospective study was based on public data from the SEER database, informed consent was not required.

### Statistical analysis

Kaplan–Meier curve and logrank tests were used to examine the OS and CSS of the EOCC patients. The objective was to identify the predictive clinical factors for OS and CSS in patients with EOCC by univariate and multivariate regression analyses. The Cox proportional hazard results were used as the basis of nomogram construction and validation. R software version 3.5.1 (http://www.R-project.org) was used for creating nomograms. A consistency index (C-index) and calibration curve were used to evaluate the performance and accuracy of the nomogram. The C-index value ranged from 0.50 to 1.00 and was positively correlated with the prediction performance of the model. This shows that the model is accompanied with a perfect discrimination ability when the value is 1.00. When the calibration curve is applied to a fully calibrated model, the prediction will fall on the 45° diagonal in the figure.

In addition, receiver operating characteristic (ROC) and curves were used to evaluate the predictive performance of nanograms, TNM stage and SEER stage. Statistical analyses were conducted using Statistical Package for the Social Sciences software (version 20.0; SPSS Inc, Chicago, IL, USA). When p < 0.05, the results were statistically significant.

In addition, receiver operating characteristic (ROC) curves were used to evaluate the predictive performance of nomograms, TNM stage, and SEER stage. Statistical analyses were conducted using the Statistical Package for the Social Sciences software (version 20.0; SPSS Inc., Chicago, IL, USA). P-values < 0.05 were considered statistically significant.

## RESULTS

### Patient baseline characteristics

In total, 10,079 eligible patients, who were diagnosed as having EOCC from 2004 to 2015, were identified in the SEER database; these cases were then randomly divided into a training set (n = 7,055) and a validation set (n = 3,024). For all patients, there were 5,123 (50.8 %) patients <37 years old and 2,487 (24.7 %) patients >40 years old. For the race group, 8,138 (75.8 %) patients were white, whereas 939 (9.3 %) patients were black. Further, 5,067 (50.3 %) were identified to be married and 4,475 (44.2 %) unmarried. With respect to TMN stage, the majority of patients were classified as N0 (8,806; 87.4 %), M0 (9,847; 97.7 %), and T1 (8,983, 89.1 %) according to laboratory examinations and postoperative pathological results. Squamous cell carcinoma was the most prevalent pathology, accounting for 60.6 % (6106) of the tumors. In the SEER stage group, 8,029 (79.7 %) patients were found to have localized disease. There were 6,191 (61.4 %) patients with a tumor size (cm) ≤ 4. The treatment protocol for the patients included chemotherapy (2,304; 22.9 %) and radiotherapy (2,724; 27.0 %). Baseline demographic and clinical characteristics of the patients are shown in Table 1.

**TABLE 1:**
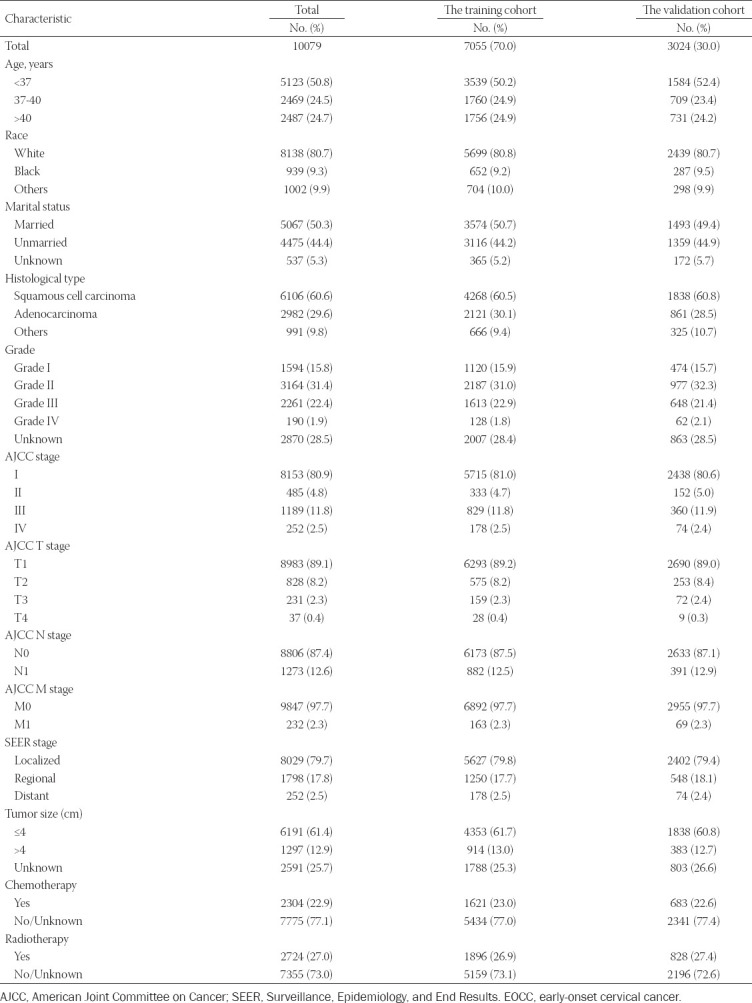
Baseline demographic and clinical characteristics with EOCC patients in our study

### Identification of independent prognostic factors of OS and CSS in training set

Univariate and multivariate Cox regression analyses were performed to identify the independent prognostic factors for OS and CSS. For the univariate analysis, these included age, race, marital status, histological type, grade, AJCC stage, T stage, M stage, N stage, SEER stage, tumor size (cm), chemotherapy, and radiotherapy as prognostic factors for OS and CSS. Meanwhile, for multivariate Cox analysis, four variables (age, marital status, N stage, and M stage) were excluded from the independent prognostic factors for OS (Table 2). The multivariate analysis also indicated that race, histological type, grade, AJCC stage, T stage, SEER stage, tumor size (cm), chemotherapy, and radiotherapy were independent prognostic factors affecting the CSS of EOCC patients (Table 3).

**TABLE 2:**
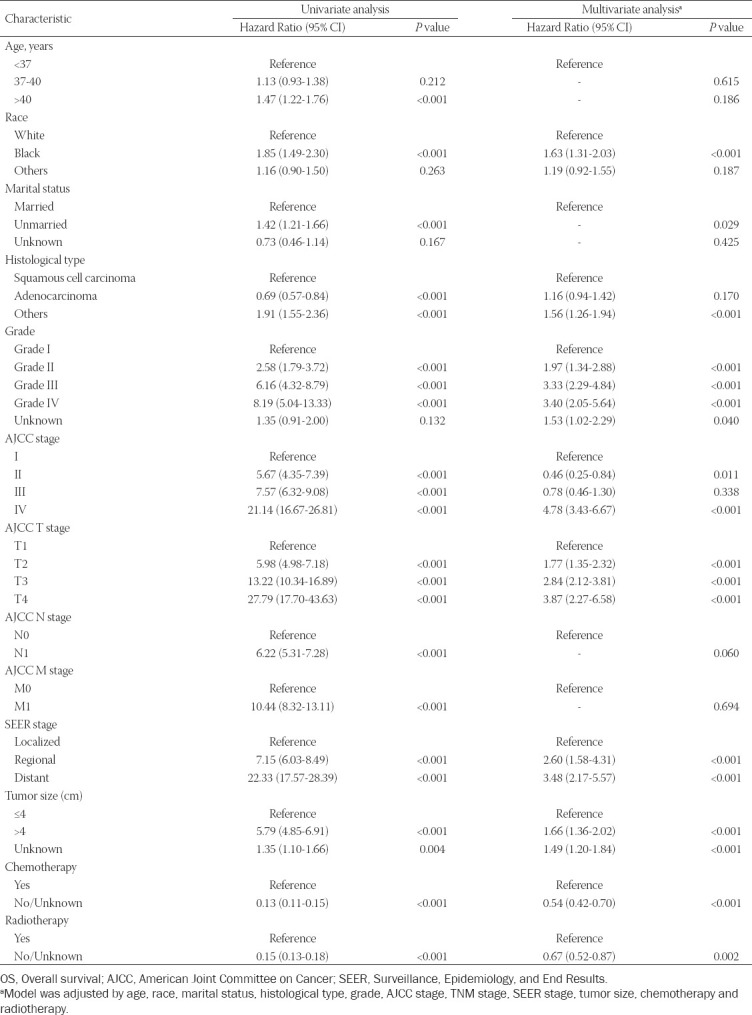
Univariate and multivariate analysis of overall survival (OS) rates in training cohort

**TABLE 3:**
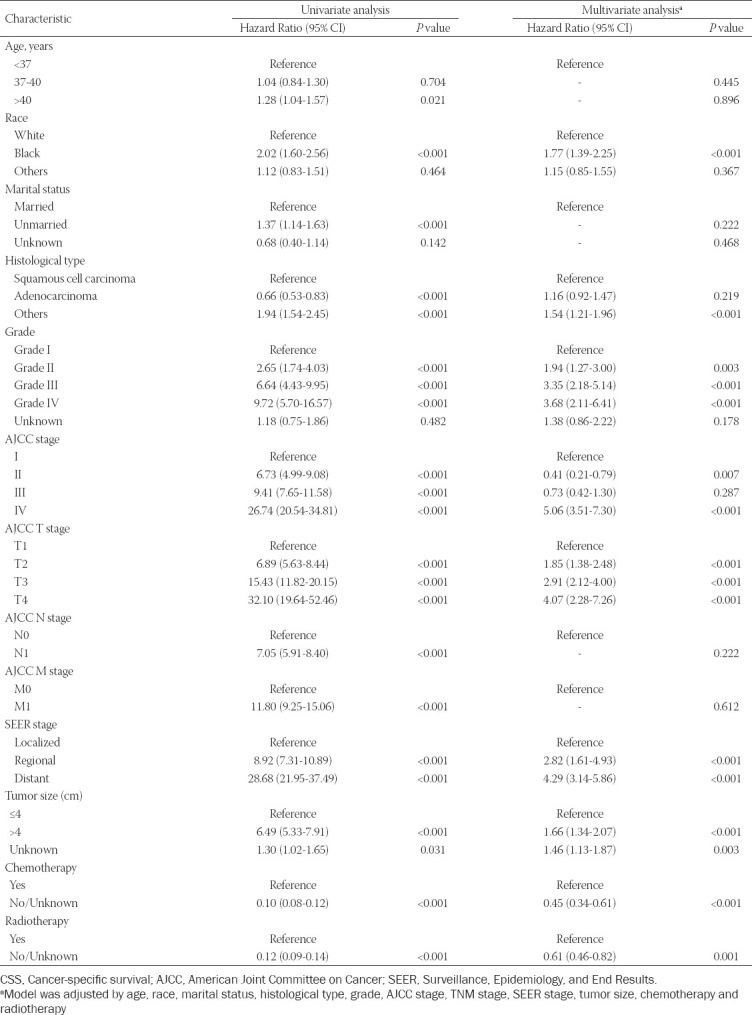
Univariate and multivariate analysis of cancer-specific survival (CSS) rates in training cohort

### Development of a prognostic nomogram for OS and CSS

**FIGURE 2:**
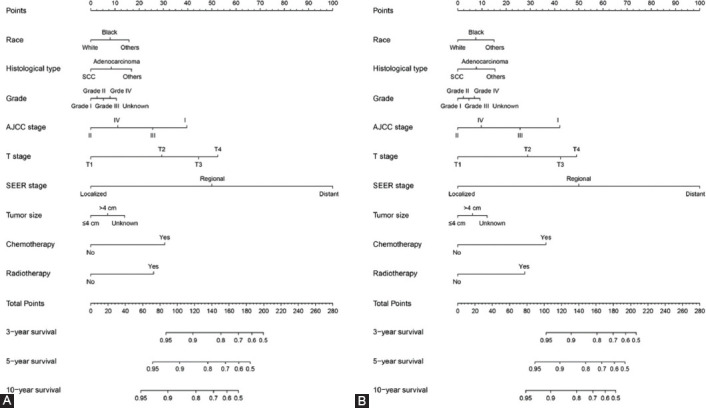
The nomogram containing independent prognostic factors for the 3-, 5-, and 10-year overall survival (OS) and cancer-specific survival (CSS) prediction of EOCC patients. A, Nomogram for OS; B, Nomogram for CSS.

The prognostic nomograms were based on the multivariate Cox regression results. The prognostic nomogram for 3-, 5-, and 10- year OS and CSS (Figs. 2A and 2B) consisted of the following independent prognostic factors: race, histological type, grade, AJCC stage, T stage, SEER stage, tumor size (cm), chemotherapy, and radiotherapy. The length of the line corresponding to each variable in the nomogram represents the contribution of the predictors to survival outcome.

Each subtype of the variables that made up the nomogram corresponds to a point on the “Points” scale. We then calculated the total score of a specific EOCC patient by adding the scores of each subtype corresponding to each variable. Then, a straight line was drawn from the position of these total scores on the “Total points” scale, thus providing each patient with 3-, 5-, and 10-year OS and CSS probabilities.

### Validation and calibration of the nomogram for OS and CSS

**FIGURE 3:**
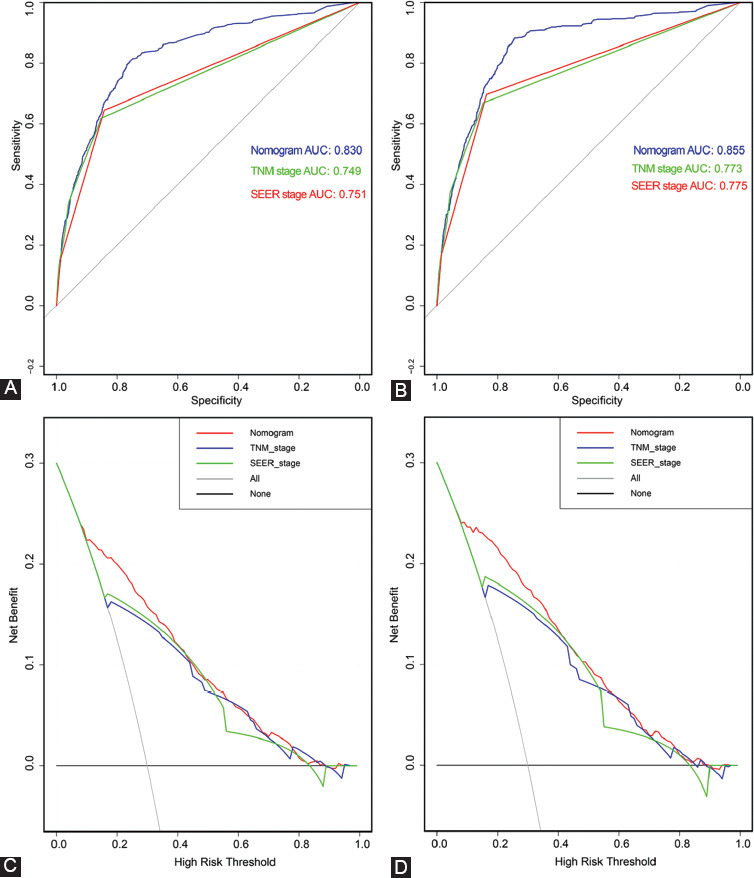
Receiver operating characteristic (ROC) and decision curve analysis (DCA) verified the predictive value of nomogram, TNM stage and SEER stage in training set. A, ROC for OS in training set. B. ROC for CSS in training set. C, DCA for OS in training set. D. DCA for CSS in training set.

**FIGURE S2:**
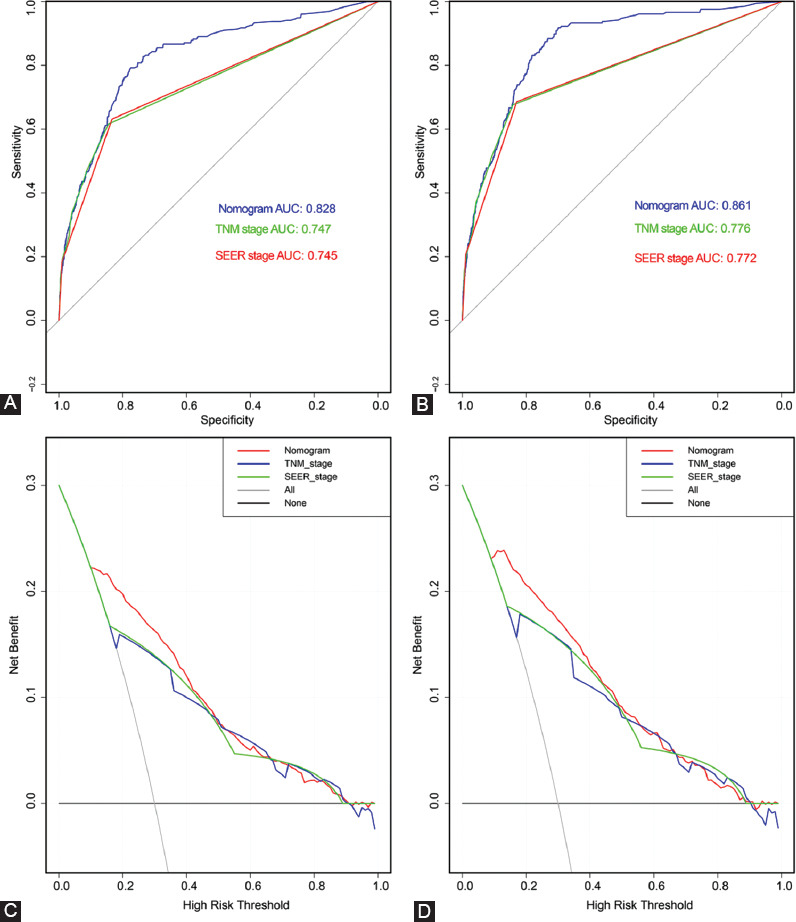
Receiver operating characteristic (ROC) and decision curve analysis (DCA) verified the predictive value of nomogram, TNM stage and SEER stage in validation set. A, ROC for OS in validation set; B. ROC for CSS in validation set; C, DCA for OS in validation set; D. DCA for CSS in validation set.

The time-dependent ROC curves for OS and CSS were used to evaluate the prediction performance of the nomogram in different sets. An AUC value of 0.5 indicates that the nomogram has no predictive effect, whereas an AUC value of 1 indicates that the nomogram can completely distinguish patients with different survival rates. The higher the value between 0.5 and 1, the stronger the resolution of the nomogram. The area under the curve (AUC) values of the nomogram for OS (Figure 3A) and CSS (Figure 3B) were 0.830 (95 % CI: 0.821–0.838) and 0.855 (95 % CI: 0.847–0.864), respectively (Table 4), in the training set, which were significantly larger than the TNM stage and SEER stage. The results were the same for the validation set. The AUCs for the nomogram were 0.828 (95 % CI: 0.814–0.842) for OS (Fig. S2A) and 0.861 (95 % CI: 0.848–0.873) for CSS (Fig. S2B). At the same time, the clinical usefulness of the nomogram was verified by DCA. The results indicated that the nomogram had a good ability for predicting OS and CSS, which was similar to the TNM stage and SEER stage in the training set (Figs. 3C, D) and validation set (Figs. S2C, D).

**TABLE 4:**
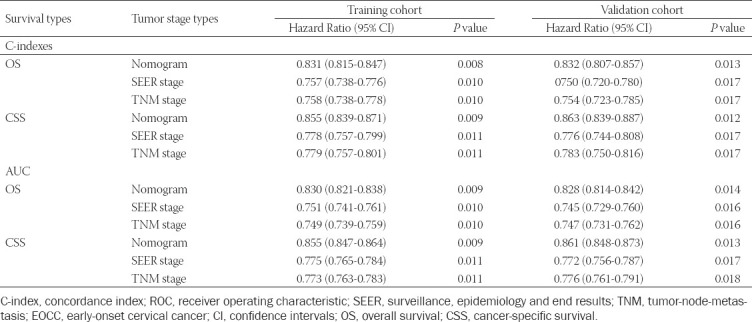
Comparison of C.indexes and AUC between the nomogram, SEER stage and TNM stage in EOCC patients

**FIGURE 4:**
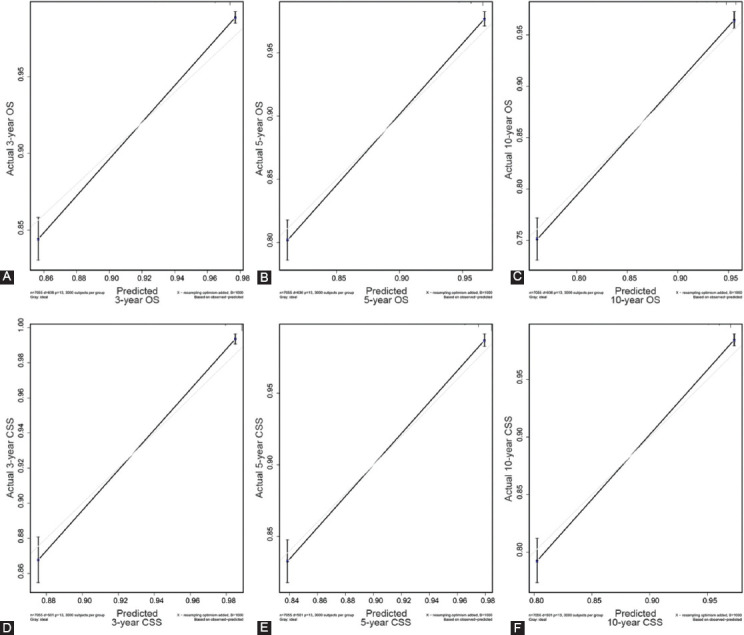
Calibration plot of the 3-, 5-, and 10-year OS and CSS nomogram in training set. A, 3-year OS in training set; B, 5-year OS in training set; C, 10-year OS in training set; D, 3-year CSS in training set; E, 5-year CSS in training set; F, 10-year CSS in training set.

**FIGURE S3:**
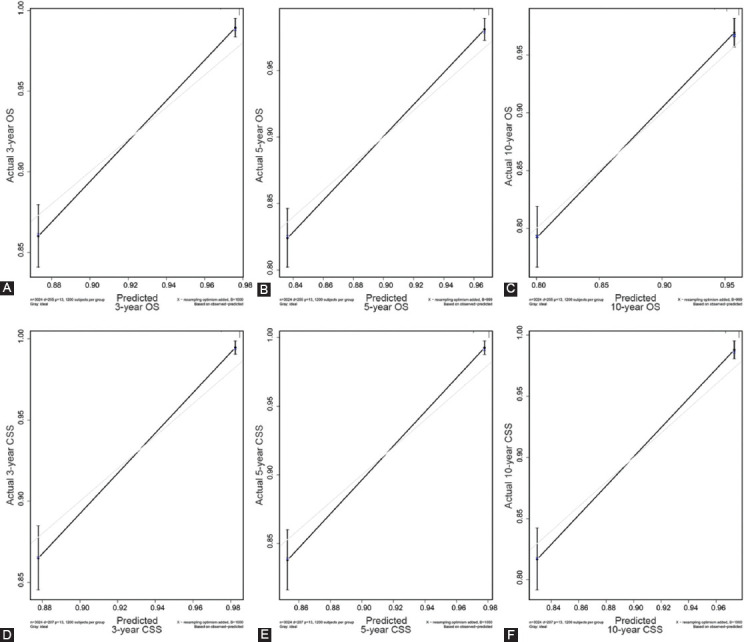
Calibration plot of the 3-, 5-, and 10-year OS and CSS nomogram in validation set. A, 3-year OS in validation set; B, 5-year OS in validation set; C, 10-year OS in validation set; D, 3-year CSS in validation set; E, 5-year CSS in validation set; F, 10-year CSS in validation set.

Next, the C-index was then used to verify the nomogram. Significant differences were noted in OS and CSS among the nomogram, TNM stage, and SEER stage (Table 4). In the training set, the C-index for OS predicted by the nomogram was 0.831 (95 % CI: 0.815–0.847), whereas the C-index for CSS was 0.855 (95 % CI: 0.839–0.871), which was higher compared with that of the TNM and SEER stages (P < 0.05). The same conclusion was drawn from the results of the validation dataset. The C-index for OS predicted by the nomogram was 0.832 (95 % CI: 0.807–0.857) and that of the CSS was 0.863 (95 % CI: 0.839–0.887) (Table 4). We have also generated a calibration curve to compare the nomogram with a perfect curve. As per the results, the 3-, 5-, and 10-year OS (Figs. 4 A,B,C) and CSS (Figs.4 D,E,F) nomograms for the training set exhibited good consistency with the actual observation, and this consistency was also evident in the validation set (Fig. S3). The calibration curves were very close to a perfect curve. The above results indicate that the predicted values of the nomogram were in good agreement with the observed values in the training and verification sets.

## DISCUSSION

Cervical cancer remains to be the fourth most common malignant tumor among women threatens the lives of most women across the globe [11]. In practice, there is a need to improve survival rates, accurately predict EOCC patient prognosis, and formulaworldwide [1,2], and its age of onset tends to be younger [10]. In addition, the mortality rate for cervical cancer ranks first among female malignant tumors, being a major disease that seriously te individualized treatment plans. Our goal is thus to develop a robust system to comprehensively consider multiple prognostic factors to accurately predict the survival time of EOCC patients.

A nomogram is a graphical representation of a multivariable prognostic model that integrates multiple prognostic factors and can be used to accurately evaluate individual probabilities of survival at certain times. This present study focused on prognosis prediction for EOCC patients based on the construction of a nomogram. First, univariate and multivariate Cox regression analysis were performed to assess independent prognostic factors for OS and CSS. The results of the multivariate analysis also indicated that race, histological type, grade, AJCC stage, T stage, SEER stage, tumor size (cm), chemotherapy, and radiotherapy were independent prognostic factors of CSS for EOCC patients. We established prognostic nomograms for 3-, 5-, and 10-year OS and CSS for EOCC patients.

The ROC curve, DCA curve, and C-index were used to verify the clinical practicability and predictive performance of the nomogram, which revealed its superiority compared with TNM stage and SEER stage. At the same time, the prediction accuracy for OS and CSS at 3-, 5-, and 10- years was evaluated using a calibration curve, which was in good agreement with the actual observational results. In practice, the area under the curve (AUC) values of the nomogram for OS (Figure 3A, S2A) and CSS (Figure 3B, S2B) were 0.830 (95 % CI: 0.821–0.838) and 0.855 (95 % CI: 0.847–0.864) in the training set and 0.828 (95 % CI:0.814–0.842) and 0.861 (95 % CI:0.848–0.873) in the validation set, respectively (Table 4). Furthermore, the C-index of OS and CSS predicted by the nomogram was 0.831 (95 % CI: 0.815–0.847) and 0.855 (95 % CI: 0.839–0.871) in the training set and 0.832 (95 % CI: 0.807–0.857) and 0.863 (95 % CI: 0.839–0.887) in the validation set, respectively (Table 4). This confirms the good predictive ability of the nomogram. The results of the DCA curve also provided proof for the robust clinical value of the nomogram.

As a prognostic tool to graphically display clinical results, a nomogram can accurately predict the overall survival (OS) and cancer-specific survival (CSS) rates of patients, which is attributed to the multiple clinical variables included in its calculation. Recently, nomograms consisting of various clinical variables have been used to predict the prognosis of different CC patients [12-15]. Wang et al. [14] analyzed cervical cancer patient data recorded in the SEER database and established a nomogram for postoperative survival prediction. It provided patients with resected CC with an accurate individualized prediction of OS, thus assisting clinicians in decision-making. Similarly, Xie et al. [15] developed a nomogram for predicting the prognosis of cervical cancer patients aged 65 years or older and comprehensively analyzed the independent prognostic factors including race, marriage, histological types, grade, FIGO, regional lymph node, surgery, radiotherapy, and chemotherapy.

In this present study, clinical variables including race, histological type, grade, AJCC stage, T stage, SEER stage, tumor size (cm), chemotherapy, and radiotherapy were independent risk factors affecting the prognosis of EOCC patients. Other studies reported that age and race are risk factors for the prognosis of various cancers [12,13,14]. Genetic differences among different races are also a significant risk factor for tumor prognosis that has been widely recognized [14,15].

The grade, tumor size, and histological type of the tumor also significantly influence patient prognosis [16,17]. In this study, these results were supported by a statistical analysis. At present, TNM stage is the most common tumor staging system in the world. It is determined by laboratory and postoperative pathological examination. However, TNM stage has limitations and does not provide an individualized prognosis prediction for a patient. In addition to TNM stage, the prognosis of patients is closely related to a variety of clinical variables. Accurate prediction depends on the consideration of all independent risk factors. We thus have successfully established an effective nomogram based on the following factors: race, histological type, grade, AJCC stage, T stage, SEER stage, tumor size (cm), chemotherapy, and radiotherapy. It proved to be a better predictive algorithm compared with TNM stage and SEER stage. The establishment of this nomogram will be useful for designing individualized treatments for EOCC patients.

Our study had some limitations. First, the SEER database did not contain detailed information regarding chemotherapy, such as the use of targeted drugs, which are important in the prognosis of CC. Because of lacking information on living environment, lifestyle, adjuvant therapy, and commodities, it was not possible to consider all prognostic factors comprehensively, which was also an intrinsic limitation of SEER research. Secondly, we did not conduct an external validation to further assess this nomogram.

## CONCLUSION

Our study is the first to construct a precise nomogram for predicting the 3-, 5-, and 10-year OS and CSS for EOCC patients, which exhibited a better predictive performance compared with TNM and SEER stages. This model may assist clinicians in designing personalized treatments for EOCC patients.

## References

[1] Siegel RL, Miller KD, Jemal A (2020). Cancer statistics, 2020. CA Cancer J Clin.

[2] Ginsburg O, Bray F, Coleman MP, Vanderpuye V, Eniu A, Kotha SR (2017). The global burden of women's cancers:a grand challenge in global health. Lancet.

[3] Gopalani SV, Janitz AE, Campbell JE (2018). Trends in cervical cancer incidence and mortality in Oklahoma and the United States, 1999–2013. Cancer Epidemiol.

[4] Arbyn M, Walker A, Meijer CJ (2010). HPV-based cervical-cancer screening in China. Lancet Oncol.

[5] Cohen PA, Jhingran A, Oaknin A, Denny L (2019). Cervical cancer. Lancet.

[6] Amin MB, Greene FL, Edge SB, Compton CC, Gershenwald JE, Brookland RK (2017). The Eighth Edition AJCC Cancer Staging Manual:Continuing to build a bridge from a population-based to a more “personalized”approach to cancer staging. CA Cancer J Clin.

[7] Touijer K, Scardino PT (2009). Nomograms for staging, prognosis, and predicting treatment outcomes. Cancer-Am Cancer Soc.

[8] Yu C, Zhang Y (2019). Development and validation of prognostic nomogram for young patients with gastric cancer. Annals of translational medicine.

[9] Pan X, Yang W, Chen Y, Tong L, Li C, Li H (2019). Nomogram for predicting the overall survival of patients with inflammatory breast cancer:A SEER-based study. Breast (Edinburgh, Scotland).

[10] Dong J, Su M, Chang W (2017). Long non-coding RNAs on the stage of cervical cancer (Review). Oncol Rep.

[11] Li J, Kang LN, Qiao YL (2011). Review of the cervical cancer disease burden in mainland China. Asian Pac J Cancer Prev.

[12] Polterauer S, Grimm C, Hofstetter G, Concin N, Natter C, Sturdza A (2012). Nomogram prediction for overall survival of patients diagnosed with cervical cancer. Br J Cancer.

[13] Wang W, Liu X, Meng Q, Zhang F, Hu K (2019). Nomograms predicting survival and patterns of failure in patients with cervical cancer treated with concurrent chemoradiotherapy:A special focus on lymph nodes metastases. PLoS One.

[14] Wang C, Yang C, Wang W, Xia B, Li K, Sun F (2018). A Prognostic Nomogram for Cervical Cancer after Surgery from SEER Database. J Cancer.

[15] Xie S, Pan S, Zou S, Zhu H, Zhu X (2020). Characteristics and Treatments of Patients Aged 65 Years or Over with Cervical Cancer. Clin Interv Aging.

[16] Intaraphet S, Kasatpibal N, Siriaunkgul S, Sogaard M, Patumanond J, Khunamornpong S (2013). Prognostic impact of histology in patients with cervical squamous cell carcinoma, adenocarcinoma and small cell neuroendocrine carcinoma. Asian Pac J Cancer Prev.

[17] Xie G, Wang R, Shang L, Qi C, Yang L, Huang L (2020). Calculating the overall survival probability in patients with cervical cancer:a nomogram and decision curve analysis-based study. BMC Cancer.

